# Ultrasound Assessment Before Complex or Difficult Cesarean Section

**DOI:** 10.3390/diagnostics16020178

**Published:** 2026-01-06

**Authors:** Kwok-yin Leung

**Affiliations:** Obstetrics and Gynaecology, Gleneagles Hospital Hong Kong, Hong Kong, China; ky@kyleung.org

**Keywords:** ultrasound, cesarean section, placenta accreta, placenta previa, fibroid

## Abstract

Complex or difficult cesareans are associated with significant short- and long-term complications. The complication rate increases with the increasing number of cesareans, and the incidence of cesarean section is increasing. To accurately identify women at high risk of surgical difficulty during a cesarean, ultrasound, in addition to clinical assessment, can be used to evaluate many risk factors, including placenta previa, placenta accreta spectrum (PAS) disorders, fibroids, severe pelvic adhesions, and membranous fetal vessels. The role of preoperative ultrasound is to identify ultrasonographic signs of anatomic changes that may affect the risk of intraoperative complications in subsequent cesarean sections. It is important to look for maternal problems as well as fetal problems. Ultrasound is a well-established practice in obstetrical care as it is easily available, accessible, easy to perform, and well accepted by women. However, there are few studies on the role of preoperative ultrasound in the management of complex or difficult cesareans beyond the risk assessment of PAS. Currently, preoperative ultrasound is mostly performed in selected cases only, with the exception in some settings. The aim of this review article is to discuss the benefits and the use of ultrasound assessment before different types of complex or difficult cesareans. Whether ultrasound assessment should be performed before all cesarean sections will also be discussed.

## 1. Introduction

Although simple elective first-time cesareans are usually safe, complex or difficult cesareans are associated with significant short- and long-term complications [1]. Over the years, the incidence of cesarean section has been increasing worldwide. The risks of placenta accreta spectrum disorder (PAS), visceral injuries, the need for postoperative ventilation, intensive care unit admission, hysterectomy, blood transfusion requiring four or more units, and the duration of operative time and hospital stay have increased with the increasing number of cesareans [2]. In particular, PAS is a life-threatening complication associated with massive postpartum hemorrhage (PPH). Another complication is severe pelvic adhesions after multiple cesareans. Other examples of complex or difficult cesareans are large uterine fibroids in the anterior lower uterine segment (LUS), and anterior major placental previa. These complex or difficult cesareans can cause major PPH, urological, or bowel injuries [1], which may require reoperation [3].

To minimize cesarean complications, the International Federation of Gynaecology and Obstetrics (FIGO) has published good practice recommendations for effective surgical techniques [4]. In complex cesareans, expert surgical skills and multidisciplinary team management are required. However, over the last two decades, obstetric doctors’ surgical training opportunities for complex cesareans were reduced because of decreased working hours, reduced number of deliveries, and the use of laparoscopy for most gynecologic operations [1].

To accurately identify women at high risk of surgical difficulty during a cesarean [1], ultrasound, in addition to clinical assessment, can be used to evaluate many risk factors, including placenta previa, PAS, fibroids, and severe pelvic adhesions [1,5]. The role of preoperative ultrasound is to identify ultrasonographic signs that indicate anatomic changes that can affect the risk of intraoperative complications in subsequent cesarean sections [5]. Specific elements to be examined depend on the suspected or confirmed disorders, such as PAS, placenta previa, or fibroids.

Before cesarean deliveries, it is important to look for maternal problems as well as fetal problems, including abnormal fetal lie or presentation, fetal growth restriction, abnormal fetal umbilical artery Doppler, oligohydramnios/polyhydramnios, or fetal anomalies. In high-risk cases such as pre-eclampsia, fetal growth restriction, or rarely thrombophilia, serial ultrasound examination and Doppler flow studies are required to assess fetal growth and well-being.

Ultrasound is a well-established practice in obstetrical care as it is easily available, accessible, easy to perform, and well accepted by women. However, there are few studies on the role of preoperative ultrasound in the management of complex or difficult cesareans beyond the risk assessment of PAS [5]. Currently, preoperative ultrasound is mostly performed in selected cases only, with the exception in only some settings. The cases are selected by the attending obstetric doctor when a disorder such as PAS or placenta previa is suspected or found.

This is a narrative review. Databases including PubMed and Google were searched by using keywords including complex or difficult or classical cesarean sections, ultrasound, and preoperative. Relevant articles, including guidelines, observational studies, and reviews published from 1 January 1980 to 31 October 2025, were included. The aim of this review article is to discuss different types of complex or difficult cesareans and the preoperative use of ultrasound assessment.

## 2. Complex or Difficult Cesarean Sections

Complex or difficult cesareans can be divided into four categories [6].

### 2.1. Abnormal Placentation

Abnormal placentation (placenta previa and PAS) and pelvic adhesions can cause bleeding during a cesarean section, although the most common cause is uterine atony [7]. In France, surgical injury during a cesarean is increasing over the years, in particular, among these women with previous cesarean and second-stage cesarean, among other characteristics [8].

Cesarean sections for placenta previa can be a complex surgery, particularly when the placenta is anterior and/or covering the internal os of the cervix because of the associated risk of massive obstetrical hemorrhage (MOH) [9]. In an anterior low-lying placenta, LUS is usually poorly formed and vascular [6].

If a patient presents with antepartum hemorrhage associated with anterior placenta previa in a subsequent pregnancy after repeated cesarean sections, the bleeding risk during subsequent emergency cesarean delivery will be very high [5]. Intraoperative problems encountered will likely include severe adhesions in the LUS, bladder, and rectus sheath, increased vascularization under the placental bed, and large areas of myometrial dehiscence [5]. PAS, if present, may cause life-threatening bleeding and injury to the urinary tract, resulting in transfusion, intensive care unit admission, cesarean hysterectomy, and other interventions [10].

### 2.2. Severe Pelvic Adhesion with Risk of Visceral Laceration or Organ Damage

Pelvic adhesions, particularly of the vesicouterine pouch, are found in more than a third of patients with a history of one or two previous cesareans [11]. Adhesions become more frequent and severe with each cumulative cesarean [12] and after postoperative wound infection [11]. Previous cesarean section, previous pelvic surgery, and the presence of adhesions are risk factors of bladder and bowel injuries as well as bleeding [7].

### 2.3. Difficult Access to the LUS

It may take a long time to extract a fetus because of difficult access to the LUS due to uterine fibroids, severe adhesion, or obesity [1]. Expert surgical skills are required to avoid or manage potential life-threatening bleeding and visceral injuries [1]. If caesarean sections are performed under general anaesthesia (GA), fetal risks will be increased with increased GA time. Furthermore, prolonged incision to delivery interval can increase the fetal risks in cases of fetal hypoxia or abnormal cardiotocography.

### 2.4. Complex Fetal Extraction

In cases of impacted fetal head, failed instrumental delivery or impacted transverse lie, difficulty in disimpacting the fetal head or delivering the fetus will be encountered [6,13]. The difficulty increases maternal complications including hemorrhage and injury to adjacent organs, as well as neonatal complications including skull fractures, brain hemorrhage, hypoxic brain injury, and, rarely, death [6,13].

In cases of velamentous cord insertion, the abnormally located fetal vessels within the membranes are prone to rupture because they are attached to the chorion without protection by Wharton’s jelly [14]. During a cesarean delivery, the membranous fetal vessels under or near the usual location of the uterine incision or LUS are at risk of being incised or torn without awareness, resulting in acute fetal blood loss, which can be life-threatening [15].

## 3. Benefits of Ultrasound Assessment Before Complex or Difficult Cesareans

### 3.1. Management in Experience Centers

Prenatal identification of PAS allows management in, or referral to, a hospital with a multidisciplinary team with expertise in complex cesareans, urinary and gastrointestinal repair, and additional resources, including transfusion services and maternal and neonatal intensive care units [5,16]. In view of the diminishing advanced surgical skills among obstetrician–gynecologists, particularly among new graduates, referring complex cesarean deliveries to experienced centers has become a common evidence-based practice [16].

According to the Royal College of Obstetricians and Gynaecologists (RCOG) guidelines, prenatal diagnosis of placenta previa allows for the arrangement of delivery in a maternity unit with on-site blood transfusion services and access to critical care [9]. Women with placenta previa and anterior low-lying placenta are at increased risk of MOH and hysterectomy. Cesarean delivery should be carried out by an appropriately experienced operator [9]. A senior obstetrician and senior anesthetist should be present for a planned delivery [9].

### 3.2. Improved Outcomes

Compared with those managed by standard obstetrical care, women with PAS managed in experience centers are less likely to require massive blood transfusion and reoperation for bleeding complications [17]. According to the International Society of Ultrasound in Obstetrics and Gynecology (ISUOG) guidelines and a systematic review, prenatal diagnosis of PAS at any gestational age is associated with improved maternal outcome, by allowing treatment in experienced centers [18,19]. Furthermore, visceral injuries (to the bladder, the ureters, and the gastrointestinal tract), if any, can be promptly diagnosed and repaired with the appropriate surgical technique or by specialist surgeons [6]. As such, better outcomes can result.

In cases of coexisting fetal problems such as fetal growth restriction or pre-eclampsia, serial preoperative ultrasound examination to assess fetal growth and well-being, among other monitoring, can help determine the optimal timing of cesarean delivery, and hence improve the neonatal outcomes.

### 3.3. Proper Surgical Plan

In complex or difficult cesareans, preoperative ultrasound assessment allows formulation of a proper surgical plan regarding the type of skin and uterine incision. Although a suprapubic transverse skin incision is usually performed for most cesareans, a subumbilical midline incision with or without upper extension is required for a classical cesarean [20].

A low transverse uterine incision is usually performed for cesarean section, with the feasibility of extending the uterine wound with T-extension (vertical) in the midline, J-extension (upward) on either side or U-extension (upward) on both sides [20]. In cases in which it is difficult to access the LUS, due to dense adhesion, PAS, or fibroids, a vertical midline uterine incision (classical cesarean section) may be needed [20].

According to expert opinion, in the case of anterior placenta previa, placental incision should be avoided, especially if PAS is suspected prenatally and engorged uterine superficial vessels are found intraoperatively. Instead, it is safer to perform a classical incision or a fundal transverse incision to deliver the fetus [20]. According to the RCOG guidelines, midline skin and/or classical uterine incisions should be considered when the fetus is in a transverse lie to avoid the placenta, particularly below 28 weeks of gestation. If the placenta is transected during the uterine incision, the umbilical cord should be clamped immediately after fetal delivery to avoid excessive fetal blood loss [9].

Rarely, a classical cesarean section may be required in cases in which it is difficult to deliver gently through a low transverse incision in (a) a backdown transverse lie of a large fetus with ruptured membranes, and impacted shoulder in the birth canal, or (b) special type of fetal anomaly, including conjoined twins, macrocrania, large sacrococcygeal teratoma, or myelomeningocele [20].

In modern obstetrics, most of the indications of classical cesarean section were placenta previa type I-IV, fibroids, poorly formed lower segment, transverse/unstable lie, and lower-segment adhesions [21].

According to expert opinion, when velamentous cord insertion is found with the membranous fetal vessels under or near the usual location of the uterine incision or LUS, extreme caution should be taken during cesarean section to avoid incising both the membranes and the fetal vessels [22]. During uterine incision, the membranes are separated gently and dissected at the vessel-free areas [22,23].

### 3.4. Proper Perioperative Management

In cases of dense pelvic adhesions, a long dissection time is required before delivering the fetus. Obstetricians need to take this into account when managing cases with an immediate threat to the life of the woman or fetus. In these cases, cesarean delivery should be performed as soon as possible, and preferably within 30 min of making the decision according to the National Institute for Health and Care and Excellence (NICE) guidelines [24].

The third-trimester scan allows for the detection of breech presentation, transverse lie, large-for-gestational age fetus, small-for-gestational age/fetal growth restriction, oligohydramnios, or polyhydramnios [25]. These abnormal findings may affect the timing and conduct of cesarean delivery, including preparation for PPH [26].

Sometimes, the indication of cesarean section, such as breech or transverse lie, may no longer be present during preoperative ultrasound assessment because of spontaneous version to cephalic presentation, and thus an unnecessary caesarean can be avoided.

A meta-analysis provides support for the accurate use of antenatal LUS measurements (myometrial or full thickness) using a standardized method in the prediction of a uterine defect (dehiscence and rupture) during trial of labor (TOL) in women with a scarred uterus. The pooled sensitivity and specificity of myometrial LUS thickness for cut-offs between 2.1 and 4.0 mm were 0.94 and 0.64, respectively. A thick LUS is less likely to have uterine rupture and is therefore suitable for a TOL, whereas a thin LUS is likely to have a uterine rupture and consequently suitable for repeat cesarean [27].

Although accurate assessments of the fetal head position, station and flexion by intrapartum ultrasound can help management of labor, there was no evidence to show an improvement in maternal, neonatal, or labor outcomes, according to a recent review [28]. Whether ultrasound findings of an impacted fetal head can help facilitate disimpaction during cesarean delivery and, hence, improve outcomes has not been studied.

## 4. Ultrasound Assessment Before Complex or Difficult Cesareans

According to the standard protocol proposed by the FIGO committee on Childbirth and PPH, before undergoing complex or difficult cesareans, all at-risk women should preferably be examined with transabdominal ultrasound, with special emphasis on the characteristics of the LUS, and the location of the placenta [1]. A transvaginal ultrasound can be added if the placenta is low-lying or previa. The sonographic signs associated with PAS, major LUS dehiscence, and severe pelvic adhesions should be assessed [5]. Other obstetric conditions, such as large anterior fibroid, large superficial vessels, or membranous fetal vessels in the LUS, fetal macrocephaly, or transverse lie, can also be detected [1].

### 4.1. PAS

According to expert opinion, screening for risk factors of PAS and assessing placental location by antenatal ultrasound are key to timely diagnosis [10]. The most common risk factor is placenta previa, diagnosed in a patient with a history of cesarean delivery. Other risk factors include a history of myomectomy, disorders of endometrial scarring, Asherman’s syndrome, uterine instrumentation, endometrial ablation, in vitro fertilization, other uterine surgeries, or multifetal pregnancy [10].

According to expert opinion, at-risk women should undergo systematic evaluation of the placenta by serial scan starting early in pregnancy [10]. The echostructure of the placenta should be evaluated in the first trimester, in particular for PAS in at-risk pregnancies [18]. Ultrasound signs suggestive of PAS disorders include low anterior implantation of the placenta/gestational sac, next to or in the scar niche, subplacental or uterovesical “hypervascularity [10,29]. Transvaginal ultrasound at the time of the late first-trimester scan can be offered to women with prior Cesarean delivery to exclude placental implantation over an exposed scar [18]. However, not all ongoing cesarean scar ectopic pregnancies in the first trimester develop into true PAS in the third trimester because some may develop into a placenta previa under a myometrial dehiscence in LUS that may involve part of the cervix [5].

According to the ISUOG guidelines and expert opinion, in the second trimester, the placental location should be defined in relation to the cervix and the area of cesarean scar(s) (low-transverse or classical) [10,30]. If the placenta overlies the anterior LUS in a woman with a prior lower segment cesarean section, the risk of PAS will be high, and a systematic evaluation for PAS by antenatal ultrasound is recommended [10,30]. Conversely, if the placenta is located far away from the level of the prior scar(s), the risk of PAS will be low.

The common signs of PAS are a loss of the clear zone (the normal hypoechoic zone between the placenta and myometrium), myometrial thinning (less than 1 mm), placental lacunae, subplacental hypervascularity, bridging vessels, placental bulging, bladder wall interruption, exophytic mass, and uterovesical hypervascularity [10]. A combination of scan findings and a woman’s a priori risk helps to determine the overall risk of PAS [10]. In women with placenta previa and clinical risk factors, the presence of abnormal signs increases the risk of PAS. However, these signs can be present in a regular placenta previa without PAS because these signs are due to LUS scarification and remodeling rather than to PAS [5]. Thus, PAS can be over-diagnosed [5].

It is difficult to define subplacental or uterovesical “hypervascularity” [29]. However, when these signs are associated with placental lacunae with large feeder vessels, these combined signs increase the risks of cesarean hysterectomy and massive transfusion, independent of the presence of PAS at delivery [31]. The prenatal evaluation of the size and vascular mapping of the suspected accreta area, including the entire LUS circulation, parametrial circulation, and existing arterial anastomoses with the bladder, may help the surgical team in planning the procedure [5].

According to the standard protocol proposed by the FIGO committee on Childbirth and PPH, prenatal ultrasonographic examination with a full bladder is required to assess PAS. The midpoint of the posterior wall of the full bladder is the reference point separating sectors S1 and S2 [1], which corresponds to the peritoneal reflection, which is found during cesarean section when the bladder is empty [1]. In contrast to sector S1, sector S2 corresponds to the lower uterine segment and cervix and receives the primary blood supply from the colpouterine arteries. Because the location of S2 is subperitoneal and retrovesical, the mobilization of the urinary bladder is required before applying low compression sutures (B-Lynch 2 or Ho-Cho) to control the bleeding due to placenta previa or PAS [1].

Recently, the traditional categorical terminology of PAS (placenta accreta, increta, and percreta) has been replaced by a descriptive grading system developed by FIGO [32] and the Society for Pediatric Pathology [33]. In particular, superficial types of PAS (placenta creta or adherent) are differentiated from subacute abruption or accessory lobes or other causes. The deep types of PAS (increta or percreta terminology) are replaced by grade 3 for all cases where the uterine wall under the placenta is thinned by >75% and qualified as to the degree of preoperative or intraoperative surgical disruption of the LUS [5]. Such differentiation is clinically relevant as cesarean hysterectomy can be avoided in selected cases of non-PAS by focal resection and repair of the LUS [5].

According to expert opinion, the absence of abnormal signs does not exclude the diagnosis; thus, PAS can be underdiagnosed [5,10]. MRI can be added in cases of inconclusive ultrasound diagnosis, severe PAS, PAS in uncommon locations, or when parametrial invasion is suspected [25].

### 4.2. Placenta Previa

Antenatal ultrasonography should be performed to determine the placental location and hence the optimal site for uterine incision [34]. According to the ISUOG guidelines, in a mid-trimester scan, the distance between the lower placental edge and the internal cervical os should be examined [30]. According to the consensus-driven practice, a transvaginal scan, requiring a separate consent, is useful to confirm the diagnosis of a low-lying placenta or placenta previa with or without PAS [35], especially in patients with high body mass index. If TVS shows a short cervical length (<3 cm) and a placenta with a thick edge (>1 cm), the risk of massive obstetrical hemorrhage and emergent cesarean hysterectomy will be increased [36]. If the distance is ≤15 mm on transvaginal scan, a follow-up examination in the third trimester is recommended. If placenta accreta is suspected, a more detailed evaluation is suggested [30].

### 4.3. Intra-Abdominal Adhesions

At present, it is not common practice to perform an ultrasound to assess intra-abdominal adhesions before all cesareans. To assess intra-abdominal adhesions, transabdominal ultrasound examination can be performed to assess the sliding of the uterus under the inner part of the rectus fascia during deep breathing [5]. ‘Sliding’ is a normal finding, whereas ‘no sliding’ is a sign of severe intra-abdominal adhesions between the uterus and rectus fascia [5]. Another sign of viscero-peritoneal adhesion is atrophy of the fascia–peritoneum complex, which shows abnormal tissue of various acoustic character and possible vascularization in color Doppler examination [37]. However, this sign is not diagnostic, as similar features may be present after a laparotomy.

In general, it is difficult to detect intraperitoneal adhesions because intestinal gases and fatty abdominal wall adversely affect ultrasound examination [38], and a very trained eye is required to detect intraperitoneal adhesions. Assessment of the intestinal loop location during special maneuvers like deep respiration, changing positions, and compressions applied by the transducer may help locate the intraperitoneal adhesions [37]. Rarely, a distended urinary bladder can be found adherent to the anterior abdominal wall.

An abdominal map consisting of nine segments can be used to document the location and extent of the adhesion. Abdominal ultrasound is accurate for diagnosing adhesions in patients undergoing repeated surgery, but is inferior to cine-MRI in detecting intra-abdominal organ adhesion [39].

### 4.4. Uterine Defect After Cesarean or Myomectomy

Uterine rupture in pregnancy is a rare and life-threatening complication. The risk of uterine rupture increases with a history of uterine surgery, such as cesarean section and abdominal or laparoscopic myomectomy [40]. Uterine dehiscence, a separation of the uterine musculature with intact uterine serosa, can be suspected on an obstetric scan [40]. Management of uterine dehiscence is difficult. According to expert opinion, planned cesarean delivery prior to the onset of labor with careful monitoring of maternal symptoms is preferred [40].

In at-risk women, performing ultrasound assessment of LUS thickness during the first trimester seems to be more informative than the second and third trimesters in one study [41]. However, as the pregnant uterus enlarges with gestation, cases of large dehiscence develop progressively [31]. Thus, first-trimester residual myometrial thickness does not correlate well with third-trimester LUS thickness in women with a previous cesarean delivery [5].

### 4.5. Uterine Fibroids

Uterine fibroids of 5 cm or larger are associated with cesarean delivery performed before labor, and the risk increases with the size of the fibroid [42]. According to expert opinion, it is important to perform the exact mapping of the anterior fibroid before cesarean section. While a uterine incision is made to deliver the fetus, the incision should not be made over a fibroid as much as possible [6].

It is controversial whether it is safe to perform myomectomy during a cesarean delivery. A meta-analysis in 2021 showed that cesarean myomectomy is associated with a clinically insignificant increase in operative time, blood loss, and hospital stay [43]. However, a subsequent meta-analysis one year later showed that cesarean myomectomies, especially of intramural fibroids, large fibroids ≥7 cm in size, and multiple fibroids, were associated with a significant risk of hemorrhage and prolonged operation duration compared to those who underwent cesarean section only [44]. According to the evidence, it may be safe to perform cesarean myomectomy by experienced surgeons using appropriate hemostatic techniques if the fibroids are not located at the cornual or close to large vessels, and in the absence of uterine atony during surgery [44]. According to a consensus opinion, careful characterization (benign fibroids or malignant uterine tumors) and location of myometrial lesions (largely subserosal or submucosal) before a myomectomy is required [45].

### 4.6. Transverse Lie

Transverse fetal lie can be easily diagnosed by an obstetric scan. Conversion of a traditional low-isthmic transverse uterine incision to an inverted-T incision may be required if there are problems in fetal extraction [6]. According to expert opinion, in multifetal pregnancy, it is important to use ultrasound to determine the number of fetuses, their lie, presentation, and the position of the placenta before cesarean section [6]. This information can facilitate the extraction of fetuses.

### 4.7. Membranous Fetal Vessels in the LUS

According to the ISUOG guidelines, it is controversial whether routine screening for velamentous cord insertion (VCI) and/or vasa previa should be performed at the mid-trimester scan because of the risk of over-diagnosing such abnormalities and implications on the resources required [30]. If umbilical cord insertion is not detected clearly by ultrasound screening during pregnancy, an abnormal cord insertion site should be suspected and searched for. Transabdominal sonography during the second trimester has a low sensitivity of 62.5%, but has a high specificity for VCI [14]. Visualization of the LUS may be obstructed by the fetal head. If VCI is found, a targeted transvaginal examination with color Doppler imaging can be considered to screen for membranous cord vessels in the LUS, depending on experience and resources [30].

### 4.8. Impacted Fetal Head

The use of intrapartum ultrasound has increased over the last two decades, but is not yet a routine practice [28,46]. Common sonographic indicators of the fetal head station include the angle of progression (AoP) and headperineum distance (HPD), which can be obtained using transperineal sonography [28,46]. Midsagittal view allows measuring the AoP, which is the angle between a line through the pubic bone’s long axis and the tangent to the deepest bony part of the fetal skull [28,46]. Axial view allows measuring the HPD, which is the shortest distance between the outer bony limit of the fetal skull and the transducer edge [28,46]. However, it is not known whether ultrasound findings of an impacted fetal head can help facilitate the disimpaction during cesarean delivery and hence reduce adverse maternal or neonatal outcomes.

## 5. Ultrasound Assessment Before All Cesarean Sections?

At present, preoperative ultrasonographic assessment, supported mainly by observational data, is not routine practice. There is a lack of studies on this issue [5]. Without any preceding obstetric ultrasound, unexpected problems like PAS, placenta previa, severe pelvic adhesions, uterine fibroids, membranous fetal vessels, or large superficial vessels in the LUS, or breech presentation may be found during subsequent cesarean section [5,9,10,15,30,42]. Rarely, PAS can be found in women without any risk factors [10]. Detection of such problems by a preoperative ultrasound can allow risk stratification and perioperative management as discussed above. However, there are disadvantages of routine preoperative ultrasound in low-risk cases, including overdiagnosis, false positives, resource utilization, and the availability of an experienced operator. Furthermore, current evidence does not demonstrate improved maternal or neonatal outcomes with routine preoperative ultrasound in low-risk cases. In my opinion, given that cesarean section is a common obstetric procedure performed in an elective or an emergent situation, it is worthwhile to consider performing an assessment of LUS and placenta localization during a commonly indicated obstetric scan. More studies on this issue are required.

According to the ISUOG guidelines, a mid-trimester fetal ultrasound examination or a third-trimester scan should include an evaluation of the placental location among other elements [25,30]. While assessing the placenta, the operator can also assess the LUS. Any uterine fibroid in the LUS can be detected as well, although formal assessment of uterine anatomy is not part of the routine obstetric scan [18,30]. The transvaginal approach is preferred to the transabdominal approach in cases of suspected posterior placenta previa [25]. A detailed ultrasound assessment should be performed to rule out PAS disorders in cases of placenta previa and prior cesarean birth or uterine surgery [25].

More than 40% of FGR cases involving stillbirths without obvious causes of FGR (or in low-risk pregnancies) were not diagnosed until after delivery [47]. The detection rate of SGA or FGR in low-risk pregnancies by serial measurement of the symphysis-fundal height is low. Thus, a routine third-trimester scan at 36 weeks’ gestation can be offered to low-risk women to improve the detection rate of late-onset FGR [48]. In a recent review, major evidence supports ultrasound screening at 36-week gestation in low-risk pregnancies to detect fetal growth abnormalities, placental disorders and other conditions, hence potentially improving maternal–fetal outcomes [49]. However, a Cochrane systematic review and meta-analysis in 2015 did not show that ultrasound performed after 24 weeks’ gestation could reduce perinatal mortality [25].

## 6. Conclusions

According to the standard protocol proposed by the FIGO committee on Childbirth and PPH, before undergoing complex or difficult cesareans, including PAS, major placenta previa, repeated cesareans, all at-risk women should preferably be examined with transabdominal ultrasound, with special emphasis on the characteristics of the LUS, and the location of the placenta [1]. A transvaginal ultrasound can be added if the placenta is low-lying or previa. The signs associated with PAS and major LUS dehiscence should be assessed [5]. Other obstetric conditions, such as anterior fibroids, membranous fetal vessels, or large superficial vessels in the LUS, fetal macrocephaly, transverse lie, and severe pelvic adhesions, can also be detected and evaluated [1,5,14,15,42]. The key ultrasound signs, associated risks and surgical implications are summarized in Table 1.

## 7. Future Direction

At present, preoperative ultrasonographic assessment, currently supported mainly by observational data, is not a routine practice for all cesarean sections. Without any obstetric ultrasound, unexpected problems like PAS, placenta previa, severe pelvic adhesions, uterine fibroids in the LUS, or breech presentation may be encountered during subsequent cesarean section [5,9,10,15,30,42]. In my opinion, given that cesarean section is a common obstetric procedure performed in an elective or an emergent situation, it is worthwhile to consider performing an assessment of LUS and placenta location during commonly indicated obstetric scans, such as the mid-trimester morphology scan and third-trimester scan [25,30]. More studies are required to evaluate such an approach. Furthermore, it is not known whether ultrasound findings of an impacted fetal head can help facilitate disimpaction during cesarean delivery and hence reduce adverse maternal or neonatal outcomes. There is a research gap. Prospective studies that evaluate whether intrapartum ultrasound alters surgical technique, delivery time, or maternal or neonatal morbidities are required.

## Figures and Tables

**Table 1:** Key ultrasound signs, associated risks, and surgical implications in complex or difficult cesarean sections.

Key Ultrasound Signs	Associated Risks	Surgical Implications
Subplacental or uterovesical “hypervascularity” associated with placental lacunae with large feeder vessels	PAS, which is a life-threatening complication with risks of massive PPH and visceral injuries	Refer to an experienced center, may require Classical CS, CH, or other measures to control PPH, and may require a specialist surgeon to repair visceral injuries
Low-lying placenta covering the cervical os, large superficial vessels in LUS	PPH and fetal risks	Experienced operator, avoids placental incision, may require classical CS and measures to control PPH
No sliding of the uterus under the inner part of the rectus fascia during deep breathing.	Severe pelvic adhesions with long IDI and risks of visceral injuries and PPH	May require classical CS or a specialist surgeon to repair visceral injuries
A separation of the uterine musculature with intact uterine serosa	Uterine rupture, a life-threatening complication; visceral injury	Planned CS before the onset of labor may require focal resection and repair of the LUS
Fibroid in the LUS	PPH	Avoid incision over fibroid and myomectomy except in selected cases; may require classical CS
Transverse lie	Fetal injuries or hypoxia	May require inverted-T uterine incision or classical CS
Membranous fetal vessels in the LUS or over cervical os	Rupture of fetal vessels, causing fetal bleeding, is a life-threatening complication	During uterine incision, avoid incising both the membranes and the fetal vessels; gently separate the membranes, and dissect at the vessel-free areas
Deeply impacted fetal head	Maternal or fetal injuries	May require special manoeuvres to facilitate disimpaction during CS

CH: Cesarean hysterectomy; CS: cesarean section; IDI: incision-delivery interval; LUS: lower uterine segment; PAS: placenta accreta spectrum disorders; PPH: postpartum hemorrhage.

## Data Availability

No new data were created or analyzed in this study. Data sharing is not applicable to this article.

## Abbreviations

The following abbreviations are used in this manuscript:

PAS	Placenta accreta spectrum disorders
LUS	Lower uterine segment
FGR	Fetal growth restriction
AoP	Angle of progression
HPD	Headperineum distance
VCI	Velamentous cord insertion
MRI	Magnetic resonance imaging
TVS	Transvaginal sonography
TOL	Trial of labor
FIGO	International Federation of Gynaecology and Obstetrics
PPH	Postpartum hemorrhage
MOH	Massive obstetric hemorrhage
GA	General anaesthesia

## References

[1] Nieto-Calvache A.J., Ramasauskaite D., PalaciosJaraquemada J.M., Hussein A.M., Jauniaux E., Eseme A., Ubom B., Rivera-Torres L.F., Nunes I., Schlembach D. (2025). Complex cesarean section: Surgical approach to reduce the risks of intraoperative complications and postpartum hemorrhage. Int. J. Gynecol. Obstet..

[2] Silver R.M., Landon M.B., Rouse D.J., Leveno K.J., Spong C.Y., Thom E.A., Moawad A.H., Caritis S.N., Harper M., Wapner R.J. (2006). Maternal morbidity associated with multiple repeat cesarean deliveries. Obstet. Gynecol..

[3] Gedikbasi A., Akyol A., Asar E., Bingol B., Uncu R., Sargin A., Ceylan Y. (2008). Relaparotomy after cesarean section: Operative complications in surgical delivery. Arch. Gynecol. Obstet..

[4] Nunes I., Nicholson W., Theron G., FIGO Childbirth and Postpartum Hemorrhage Committee (2023). FIGO good practice recommendations on surgical techniques to improve safety and reduce complications during cesarean delivery. Int. J. Gynecol. Obstet..

[5] Jauniaux E., Fox K.A., Einerson B., Hussein A.M., Hecht J.L., Silver R.M. (2023). Perinatal assessment of complex caesarean delivery: Beyond placenta accreta spectrum. Am. J. Obstet. Gynecol..

[6] Visconti F., Quaresima P., Rania E., Palumbo A.R., Micieli M., Zullo F., Venturella R., Carlo C.D. (2020). Difficult caesarean section: A literature review. Eur. J. Obstet. Gyneco. Reprod. Biol..

[7] Field A., Haloob R. (2016). Complications of caesarean section. Obstet. Gynaecol..

[8] de Vries P.L.M., Verspyck E., Morau E., Saucedo M., Deneux-Tharaux C. (2024). on behalf of the ENCMM study group. Maternal mortality due to obstetric hemorrhage by surgical injury during cesarean section: A nationwide study. Acta Obstet. Gynecol. Scand..

[9] Jauniaux E., Alfirevic Z., Bhide A.G., Belfort M.A., Burton G.J., Collins S.L., Dornan S., Jurkovic D., Kayem G., Kingdom J. (2019). Placenta praevia and placenta accreta: Diagnosis and management: Green-top Guideline No. 27a. BJOG Int. J. Obstet. Gynaecol..

[10] Einerson B.D., Gilner J.B., Zuckerwise L.C. (2023). Placenta Accreta Spectrum. Obstet. Gynecol..

[11] Moro F., Mavrelos D., Pateman K., Holland T., Hoo W.L., Jurkovic D. (2015). Prevalence of pelvic adhesions on ultrasound examination in women with a history of cesarean section. Ultrasound Obstet. Gynecol..

[12] Lyell D.J. (2011). Adhesions and perioperative complications of repeat cesarean delivery. Am. J. Obstet. Gynecol..

[13] Alexander J.M., Leveno K.J., Hauth J., Landon M.B., Thom E., Spong C.Y., Varner M.W., Moawad A.H., Caritis S.N., Harper M. (2006). Fetal injury associated with cesarean delivery. Obstet. Gynecol..

[14] Buchanan-Hughes A., Bobrowska A., Visintin C., Attilakos G., Marshall J. (2020). Velamentous cord insertion: Results from a rapid review of incidence, risk factors, adverse outcomes and screening. Syst. Rev..

[15] Jain V., Gagnon R. (2023). Guideline No. 439: Diagnosis and Management of Vasa Previa. J. Obstet. Gynaecol. Can..

[16] Futterman I.D., Conroy E.M., Chudnoff S., Alagkiozidis I., Minkoff H. (2024). Complex obstetrical surgery: Building a team and defining roles. Am. J. Obstet. Gynecol. MFM.

[17] Bartels H.C., Rogers A.C., O’Brien D., McVey R., Walsh J., Brennan D.J. (2018). Association of Implementing a multidisciplinary team approach in the management of morbidly adherent placenta with maternal morbidity and mortality. Obstet. Gynecol..

[18] Bilardo C.M., Chaoui R., Hyett J.A., Kagan K.O., Karim J.N., Papageorghiou A.T., Poon L.C., Salomon L.J., Syngelaki A., International Society of Ultrasound in Obstetrics and Gynecology (2023). ISUOG Practice Guidelines (updated): Performance of 11–14-week ultrasound scan. Ultrasound Obstet. Gynecol..

[19] Timor-Tritsch I., Buca D., Di Mascio D., Cali G., D’Amico A., Monteagudo A., Tinari S., Morlando M., Nappi L., Greco P. (2021). Outcome of cesarean scar pregnancy according to gestational age at diagnosis: A systematic review and meta-analysis. Eur. J. Obstet. Gynecol. Reprod. Biol..

[20] Kan A. (2020). Classical Cesarean Section. Surg. J..

[21] Hui W., Ng V.K.S., LAU W.M., Leung W.C. (2017). Classical Caesarean Section Revisited in Modern Obstetrics. Hong Kong J. Gynaecol. Obstet. Midwifery.

[22] Oyelese Y., Iammatteo M., Domnitz S., Chavez M.R. (2022). Vasa previa: Avoiding incising the membranes at cesarean delivery. Am. J. Obstet. Gynecol..

[23] Sairem M.C., Papa D., Chitra T., Bhabani P., Rajalakshmi T. (2024). Prenatal Ultrasound Diagnosis of Vasa Previa with Careful Intraoperative Management: A Case Report. Cureus.

[24] National Institute for Health and Care and Excellence (NICE) Caesarean Birth. *NICE* guideline March 2021. https://www.nice.org.uk/guidance/NG192.

[25] Khalil A., Sotiriadis A., D’Antonio F., Da Silva C.F., Odibo A., Prefumo F., Papageorghiou A.T., Salomon L.J. (2024). ISUOG Practice Guidelines: Performance of third-trimester obstetric ultrasound scan. Ultrasound Obstet. Gynecol..

[26] Mavrides E., Allard S., Chandraharan E., Collins P., Green L., Hunt B.J., Riris S., Thomson A.J., on behalf of the Royal College of Obstetricians and Gynaecologists (2016). Prevention and management of postpartum haemorrhage. BJOG.

[27] Kok N., Wiersma I.C., Opmeer B.C., De Graaf I.M., Mol B.W., Pajkrt E. (2013). Sonographic measurement of lower uterine segment thickness to predict uterine rupture during a trial of labor in women with previous Cesarean section: A meta-analysis. Ultrasound Obstet. Gynecol..

[28] Zegarra R.R., Cepeda E.L., Tullio Ghi T. (2025). Intrapartum ultrasound. Best. Pract. Res. Clin. Obstet. Gynaecol..

[29] Jauniaux E., D’Antonio F., Bhide A., Prefumo F., Silver R.M., Hussein A.M., Shainker A.S., Chantraine F., Alfirevic Z., Delphi Consensus Expert Panel (2023). Modified Delphi study of ultrasound signs associated with placenta accreta spectrum. Ultrasound Obstet. Gynecol..

[30] Salomon L.J., Alfirevic Z., Berghella V., Bilardo C.M., Chalouhi G.E., Da Silva Costa F., Hernandez-Andrade E., Malinger G., Munoz H., Paladini D. (2022). ISUOG Practice Guidelines (updated): Performance of the routine mid-trimester fetal ultrasound scan. Ultrasound Obstet. Gynecol..

[31] Einerson B.D., Comstock J., Silver R.M., Branch D.W., Woodward P.J., Kennedy A. (2020). Placenta accreta spectrum disorder: Uterine dehiscence, not placental invasion. Obstet. Gynecol..

[32] Jauniaux E., Ayres-de-Campos D., LanghoffRoos J., Fox K.A., Collins S. (2019). FIGO Placenta Accreta Diagnosis and Management Expert Consensus Panel. FIGO classification for the clinical diagnosis of placenta accreta spectrum disorders. Int. J. Gynaecol. Obstet..

[33] Hecht J.L., Baergen R., Ernst L.M., Katzman P.J., Jacques S.M., Jauniaux E., Khong T.Y., Metlay L.A., Poder L., Qureshi F. (2020). Classification and reporting guidelines for the pathology diagnosis of placenta accreta spectrum (PAS) disorders: Recommendations from an expert panel. Mod. Pathol..

[34] Boehm F.H., Fleischer A.C., Barrett J.M. (1982). Sonographic placental localization in the determination of the site of uterine incision for placenta previa. J. Ultrasound Med..

[35] Reddy U.M., Abuhamad A.Z., Levine D., Saade G.R. (2014). Fetal Imaging Workshop Invited Participants. Fetal imaging: Executive summary of a joint Eunice Kennedy Shriver National Institute of Child Health and Human Development, Society for Maternal-Fetal Medicine, American Institute of Ultrasound in Medicine, American College of Obstetricians and Gynecologists, American College of Radiology, Society for Pediatric Radiology, and Society of Radiologists in Ultrasound Fetal Imaging Workshop. J. Ultrasound Med..

[36] Zaitoun M.M., El Behery M.M., Abd El Hameed A.A., Soliman B.S. (2011). Does cervical length and the lower placental edge thickness measurement correlates with clinical outcome in cases of complete placenta previa?. Arch. Gynecol. Obstet..

[37] Smereczyński A., Starzyńska T., Kołaczyk K., Bojko S., Gałdyńska M., Bernatowicz E., Walecka A. (2013). Intra-abdominal adhesions in ultrasound. Part II: The morphology of changes. J. Ultrason..

[38] Lienemann A., Sprenger D., Steitz H.O., Korell M., Reiser M. (2000). Detection and mapping of intraabdominal adhesions by using functional cine MR imaging: Preliminary results. Radiology.

[39] Yasemin A., Mehmet B., Omer A. (2020). Assessment of the diagnostic efficacy of abdominal ultrasonography and cine magnetic resonance imaging in detecting abdominal adhesions: A double-blind research study. Eur. J. Radiol..

[40] Whittington J.R., Slaton K.B., Rhomberg M.E., Ghahremani T., Thomas S.L., Magann E.F. (2021). Uterine Dehiscence and Subsequent Pregnancy Management: A Review of the Literature. Obstet. Gynecol. Surv..

[41] Spahn S., Horky A., Sugiyo D., Bahlmann F., Naimi A.A. (2025). The prospective sonographic assessment of the lower uterine segment after cesarean section and its clinical utility. Arch. Gynecol. Obstet..

[42] Vergani P., Locatelli A., Ghidini A., Andreani M., Sala F., Pezzullo J.C. (2007). Large uterine leiomyomata and risk of cesarean delivery. Obstet. Gynecol..

[43] Goyal M., 1Ayman Shehata Dawood A.S., Elbohoty S.B., Abbas A.M., Singh P., Melana N., Singh S. (2021). Cesarean myomectomy in the last ten years; A true shift from contraindication to indication: A systematic review and meta-analysis. Eur. J. Obstet. Gynecol. Reprod. Biol..

[44] Huang Y., Ming X., Li Z. (2022). Feasibility and safety of performing cesarean myomectomy: A systematic review and meta-analysis. J. Matern. Fetal Neonatal Med..

[45] den Bosch V., Dueholm M., Leone F.P.G., Valentin L., Rasmussen C.K., Votino A., Van Schoubroeck D., Landolfo C., Installé A.J.F., Guerriero S. (2015). Terms, definitions and measurements to describe sonographic features of myometrium and uterine masses: A consensus opinion from the Morphological Uterus Sonographic Assessment (MUSA) group. Ultrasound Obstet. Gynecol..

[46] Dall’Asta A., Melito C., Ghi T. (2024). Intrapartum Ultrasound Guidance to Make Safer Any Obstetric Intervention: Fetal Head Rotation, Assisted Vaginal Birth, Breech Delivery of the Second Twin. Clin. Obstet. Gynecol..

[47] Wong S.T.K., Tse W.T., Lau S.L., Sahota D.S., Leung T.Y. (2022). Stillbirth rate in singleton pregnancies: A 20-year retrospective study from a public obstetric unit in Hong Kong. Hong Kong Med. J..

[48] Leung K.Y. (2025). Perinatal deaths in singleton pregnancy in Hong Kong. Hong Kong J. Gynaecol. Obstet. Midwifery.

[49] Emam D., Corbella G., Poziello C., Fabozzo S., Farina A., Candiani M., Kagan K.O., Cavoretto P.I. (2025). Usefulness and timing of the third-trimester ultrasound scan: A review of guidelines and underlying evidence. Arch. Gynecol. Obstet..

